# Childhood Trauma and Personal Mastery: Their Influence on Emotional Reactivity to Everyday Events in a Community Sample of Middle-Aged Adults

**DOI:** 10.1371/journal.pone.0121840

**Published:** 2015-04-07

**Authors:** Frank J. Infurna, Crystal T. Rivers, John Reich, Alex J. Zautra

**Affiliations:** Department of Psychology, Arizona State University, Tempe, Arizona, United States of America; National Center of Neurology and Psychiatry, JAPAN

## Abstract

Childhood trauma is associated with premature declines in health in midlife and old age. Pathways that have been implicated, but less studied include social-emotional regulation, biological programming, and habitual patterns of thought and action. In this study we focused on childhood trauma’s influence via alterations in social-emotional regulation to everyday life events, a pathway that has been linked to subsequent health effects. Data from a 30-day daily diary of community residents who participated in a study of resilience in Midlife (*n* = 191, *M_age_* = 54, *SD* = 7.50, 54% women) was used to examine whether self-reports of childhood trauma were associated with daily well-being, as well as reported and emotional reactivity to daily negative and positive events. Childhood trauma reports were associated with reporting lower overall levels of and greater variability in daily well-being. Childhood trauma was linked to greater reports of daily negative events, but not to positive events. Focusing on emotional reactivity to daily events, residents who reported higher levels of childhood trauma showed stronger decreases in well-being when experiencing negative events and also stronger increases in well-being with positive events. For those reporting childhood trauma, higher levels of mastery were associated with stronger decreases in well-being with negative events and stronger increases in well-being with positive events, suggesting that mastery increases sensitivity to daily negative and positive events. Our results suggest that childhood trauma may lead to poorer health in midlife through disturbances in the patterns of everyday life events and responses to those events. Further, our findings indicate that mastery may have a different meaning for those who experienced childhood trauma. We discuss social-emotional regulation as one pathway linking childhood trauma to health, and psychosocial resources to consider when building resilience-promoting interventions for mitigating the detrimental health effects of childhood trauma.

## Introduction

Development is a lifelong process with experiences from childhood potentially having an impact on health and well-being throughout in midlife and beyond [1–3]. Childhood trauma characterized by abuse and family conflict is one of those early life experiences that not only has detrimental effects during childhood and adolescence, but can leave a “scar” well into midlife and old age [4–5]. Recent empirical evidence suggests that childhood trauma is associated with less emotional support and more strain in social relationships in adult life [6], lower levels of well-being [7], and early onset of functional limitations, disease, and premature mortality [8–10].

There are several possible pathways linking childhood trauma to health in midlife, including social-emotional regulation, biological programming, and patterns of behavior [4, 11]. For example, chronic stress in childhood is associated with stronger pro-inflammatory cytokine response and resistance to anti-inflammatory properties of cortisol [12], which have long-term health consequences [13]. Similarly, chronic childhood stress is linked to unhealthy habits, such as smoking, alcohol dependency, and overeating [14, 15]. One likely pathway that has yet to be fully tested is whether childhood trauma alters day-to-day life experiences through daily well-being, and experience of and reactivity to daily negative and positive events. These components of daily life are considered a form of social-emotional regulation that has the potential to accumulate over the lifespan to shape the course of development [16–19]. As is accustomed in the daily diary literature, we define well-being as one’s level of negative and positive affect on days when no negative or positive daily event is reported [16, 19]. Social-emotional regulation is broadly defined as one’s ability to effectively manage their daily emotions in the response to specific stimuli [20]. We conceptualize social-emotional regulation in our study as changes in positive and negative affect in response to the stimulus provided by daily negative and positive events. There may be psychosocial resources that moderate one’s social-emotional regulation capacities; we examine whether mastery, a key resilience resource across the life-span, increases or decreases social-emotional regulation capacities for individuals who experienced high levels of childhood trauma. To evaluate associations between childhood trauma and social-emotional regulation, we used data from 30-day daily diaries of participants in midlife to examine (1) whether childhood trauma is associated with daily well-being, and reports of and reactivity to daily negative and positive events and (2) the role of mastery in moderating such associations.

### Pathways Linking Childhood Trauma to Health

Childhood trauma in the form of emotional, physical, or sexual abuse, as well as childhood misfortune can have detrimental and long-lasting effects on development across the lifespan. For example, a meta-analysis by Wegman and Stetler [21] found that the effect size linking childhood abuse to negative physical health outcomes in adulthood was *d* = 0.42 (Confidence Interval = 0.39–0.45). Similarly, Caspi and colleagues [22–23] found that child maltreatment and traumatic events early in life were associated with lower well-being and greater psychological distress in young adulthood. Despite the substantial evidence linking childhood trauma to health in midlife and old age, much less empirical research has been done on examining specific pathways that could underlie this relationship.

The ramifications of childhood trauma for development in midlife and old age are thought to be set in motion through biopsychosocial processes that unfold over time to increase risk for ill health. Miller and colleagues [4] reviewed evidence for several pathways, including biological programming, behavioral habits, social relationships, and social-emotional regulation (reports of and reactivity to stressors), as plausible mechanisms. We focus on exploring social-emotional regulation as one pathway through which childhood trauma can lead to differential health outcomes because childhood trauma has the potential to affect the dynamics of daily life. Day-to-day living accumulates to affect development across the lifespan through various sets of experiences, interactions, and events. For example, Charles and colleagues [24] found that people who were more reactive to daily stressors (i.e., stronger decline in well-being) had an increased risk for mental health disorders over 10 years of time. One might describe these experiences as being comprised of multiple components, which we have captured through use of daily diaries completed by a mid-life sample. The diary data allows us to investigate levels of and variability in daily well-being, reports of both stressors and pleasant events, and emotional reactivity to daily negative and positive events. Well-being has many definitions in the literature, but a common feature to most of those definitions is attention to both the presence of positive affective states and the relative absence of negative emotions [25–27]. Following in this vein we operationalized well-being as according to one’s daily levels of and variability in negative and positive affect, in addition to how negative and positive affect changes in response to daily negative and positive events. Below we set forth the assumptions upon which our investigation is based.

First, childhood trauma may be associated with reporting lower overall levels of well-being and greater fluctuations from day-to-day (i.e., variability). Childhood trauma could alter individuals’ strategies for regulating their desires and emotions that are essential for interpreting and experiencing their daily lives in context [28–29]. Second, childhood trauma may lead to poorer health via reporting more daily stressful events and fewer daily positive events. Daily events may be more likely to be appraised and perceived as stressful and high in severity, due to inconsistencies and growing up in an environment where behavior-event relationships were not developed due to a harsh childhood environment, e.g., contingency[30]. Childhood trauma could also result in being less engaged in goal-directed behaviors that are associated with daily positive events, resulting in deriving less benefit from these events in daily life. Third, childhood trauma may prime individuals to be more sensitive to their daily events or context; this is conceptualized as one’s emotional reactivity and assessed via examining changes in well-being on days where daily negative and positive events are reported. In adolescence, childhood maltreatment is associated with poor self-regulation deficits when encountering social stressors [31], suggesting that deficits are already present early in life and likely worsen into adulthood. Similarly, previous research in adulthood shows that early life adversity and poor parental relationship quality is associated with stronger increases in negative affect to daily stressors [32–34].

In sum, there are multiple social-emotional regulation pathways through which childhood trauma may play a role in creating self-regulation deficits. Accordingly, childhood trauma may lay the groundwork for such deficits extending into midlife and beyond. These sorts of outcomes indicate that such inadequate regulatory response to stress compromises resilience.

#### The experience of well-being

Early life adversity may lead to increased sensitivity to ongoing stressors, but also differentially boost well-being through greater responsiveness to lasting changes that have a positive valence. This concept has been referred to in the literature as differential susceptibility, which is broadly defined as individual differences in one’s response to both negative and positive social contexts [8, 35]. More specifically, not only does differential susceptibility refer to a heightened reactivity to adversity, “these same attributes that make an individual particularly sensitive to adversity may also make them more responsive to interventions designed to offset the effects of adversity” [8].

One way to assess differential susceptibility is by assessing individuals’ emotional reactivity to daily social stressors and positive interactions. Despite the difficulties experienced early in life, positive daily events may provide individuals the opportunity to be engaged in their daily life and pursue goal-directed behavior, resulting in the opportunity to flourish. Most of the literature examining emotional reactivity in the context of childhood trauma has primarily focused on daily stressors, whereas *emotional reactivity to daily positive events* has been rarely addressed [19]. Positive events, such as completing a fulfilling project at work or a social interaction with family member or friend may boost well-being differentially with those who experienced childhood trauma because of being more sensitive to their context.

### Mastery as a Key Resilience Factor for Confronting Childhood Trauma

The evidence provided by prior research makes clear the potential for negative consequences arising from childhood trauma. However, there is a resilience perspective on childhood adversity that is instructive insofar as it gives voice to the potential for abused persons to recover, sustain their sense of purpose and even thrive [36]. Masten [37] described the need for researchers to examine more than the effects of deficits such as early abuse; rather, to investigate how “assets, risks, and protective factors… may influence each other over time.” Positive influences include access to caring adults, cognitive skills, and personal mastery [37, 38]. In the present study, we focus on one of these personal assets as a protective factor for individuals who experienced childhood trauma: personal mastery.

#### Personal mastery

Mastery, or perceived control, has a long history in the literature of being associated with better cognitive, mental, and physical health across the lifespan [39–42] and being a resource for caregivers and patient populations to protect against declines as a function of chronic stressors [43–44]. For our purposes, mastery is considered a resource for individuals with childhood trauma to protect against reports of and emotional reactivity to daily negative events, and could also result in positive events being more uplifting (i.e., increase in well-being). Previous research in daily diary designs has shown that mastery is protective against declines in well-being as a function of daily stressors [45]. For example, Hay and Diehl [46] found that reporting higher levels of control on stressor days was associated with a less steep increase in negative affect [47]. Mastery likely serves this protective function through individuals’ perceptions of higher control over the situation, using better coping or compensatory strategies, and turning to social network members to help reduce the negative effects of stressors. In contrast, much less is known regarding mastery in the context of daily positive events. Reporting more mastery may result in more uplifting or boosts in well-being with daily positive events because these events are often times engaged in or directed by the individual [19]. Insights on the role of mastery in the context of daily positive events may be gained through considering research on agency and self-efficacy. Bandura described self-efficacy, people’s beliefs about their ability to control events, as a mechanism of agency, which may be thought of as goal-directed determination and motivation [48]. In the context of childhood trauma, a greater sense of agency might be highly stabilizing, resulting in greater reactivity with positive events.

The view of oneself as the effective agent of one’s own life, once established and reinforced by lengthy and diverse experience, is carried forward into late life as a powerful influence on the current mastery of older adults [49]. However, in the context of childhood trauma, the role of mastery is less clear. We envision several scenarios regarding whether mastery will up- or down-regulate reports of and emotional reactivity to daily negative and positive events. Perceiving control over life can be potentially beneficial given early life adversity. For daily negative events, following previous research [45, 47], we expect that higher levels of mastery would be protective against declines in well-being when confronted with a daily negative event [50]. However, adults with high mastery may expect to see their world as under their control and daily stressors can effectively disconfirm those expectations, possibly giving rise to distress. Focusing on positive daily events, high mastery may result in stronger increases in well-being because positive events are often sought after and perceived as under one’s own control [19]. The person is an active agent rather than a passive recipient in the production of positive events [51–52] and this is amplified in the context of childhood trauma.

### The Present Study

Early life adversity in the form of childhood trauma can have a long-term impact on health in midlife and beyond; we focus on whether childhood trauma is associated with social-emotional regulation as one potential pathway underlying this association. To do so, we use data from a 30-day daily diary of participants in midlife that permits the opportunity to examine within-person change in daily well-being and tracking within-person change in well-being as a function of daily negative and positive events.

Our first objective is to examine whether childhood trauma is associated with levels and variability of daily well-being, and reports of and emotional reactivity to daily negative and positive events. We hypothesize that childhood trauma will be associated with reporting lower levels of, and greater variability in, daily well-being. Furthermore, childhood trauma will be linked to reporting more daily negative events and less engagement in daily positive events, but stronger emotional reactivity to both daily negative and positive events. Our second research question centers on the role of mastery moderating reports of and emotional reactivity to daily negative and positive events. We hypothesize that mastery will be protective against declines in well-being with daily stressors for people with high levels of childhood trauma and stronger increases in well-being as a function of daily positive events.

## Method

### Participants and Procedure

We used data from the ASU Live Project, which is a large-scale study of mid-aged (aged 40–65) residents of the Phoenix metro area (*N* = 800) focusing on identifying individual, familial and community factors in resilience (Resilience Processes in Individuals and Communities: R01 AG26006). A total of 800 participants were recruited for the study, with a final number of 782 participating in the initial component of the study that involved self-report questionnaires. The study was multi-modal. Participants completed self-report questionnaires about early family life, personality, traumatic and stressful events, as well as qualitative interviews about participants’ most stressful life experience. One-quarter of the sample (~200) participated in an additional videotaped lab stressors component and one-quarter (~200) completed daily diaries covering a 30-day period. The laboratory stressor employed procedures for eliciting physiological and affective arousal [53]. Participants were given a stress-eliciting task that was videotaped and ratings of positive and negative affect were collected with the PANAS [27] at baseline and after each rest and task period. Saliva samples (for cortisol) were collected at baseline and following each rest and stress period.

We use data from 191 participants who completed the 30-day daily diary and provided data on our variables of interest from the self-report questionnaires. On average, participants were 54 years of age (*SD* = 7.45, range 40–65), 54% were women, and 75% attended some college. The data used for the present study can be found in S1 Dataset.

#### Sampling and Recruitment

The parent study employed a purposive sampling strategy, also referred to as sampling for heterogeneity [54, 55], to recruit eight hundred participants from 40 census tracts across the metropolitan Phoenix area between the years 2007 and 2012. This method increased the external validity of research findings through representativeness of individuals, environment and measured outcomes. Census tracts were established through a factor analysis that yielded five dimensions that together explain 80% of the variance between census tracts. The five dimensions are: (1) social status (describing income, occupation, and education); (2) the presence of school-aged children and multi-person households; (3) retirement communities; (4) residential construction growth between 1995 and 2000; and (5) Native American communities.

Potential participants were contacted in two ways: (1) through mailed recruitment letters printed in both English and Spanish, and (2) recruiters traveled to households approximately one week after letters were mailed to introduce themselves and the study, provide materials about the study, and request participation. Prior to participation, participants gave informed consent. Participants were compensated up to one hundred US dollars for participation in the main study (i.e., questionnaires, home visit, phone interview), and the 25% that was selected for the lab stressors and daily diaries were compensated up to an additional one hundred and forty US dollars. Inclusion criteria for recruitment were: (1) participant presently between the ages of 40 and 65 years, and (2) either English or Spanish speaking. Exclusionary criteria were: presence of physical, psychiatric or cognitive impairments during initial recruitment contact, as measured by the Mental Status Questionnaire [56]. No participants were excluded based on these criteria. Attempts were made to keep balance between genders. The study was approved by the Arizona State University Institutional Review Board. Prior to participation, participants gave written informed consent.

#### Daily diaries

The daily diaries collected provided accounts of participants’ daily life events near in time to the events, as they occurred. Participants were given PC tablets pre-loaded with structured questions that related to the day’s most positive and negative events, and questions designed to gauge affect. They were instructed to complete the diary each evening for 30 consecutive days, 30 minutes prior to going to sleep.

### Measures

#### Childhood trauma

We used the childhood trauma questionnaire (CTQ) to assess the degree to which individuals experienced trauma in childhood [57]. The CTQ is a retrospective report that assesses the degree to which individuals experienced emotional, physical, and sexual abuse before the age of 18 (*M* = 1.68, *SD* = 0.86, range: 1–4.8; α = 0.92). Items were answered on a 5-point Likert scale, ranging from 1 (*never true*) to 5 (*very often true*). Using the stem question, “when I was growing up” participants responded to 10 items. Sample items include “People in my family called me things like “stupid,” “lazy,” or “ugly;” “I believe that I was physically abused,” and, “Someone molested me.” Although the CTQ is a retrospective report, it has been used in clinical populations for guidelines for determining whether people reported significant emotional, physical, and sexual abuse during childhood [58]. We created a mean score across the ten items that assessed emotional, physical, and sexual abuse, with higher scores reflecting higher levels of childhood trauma. The CTQ was given in the self-report questionnaire prior to participants completing the 30-day diary.

#### Mastery

Mastery was assessed using the Pearlin Mastery Scale, α = .82 [43]. Items asked participants to rate the extent to which they believe their life is under their own control (“I can do just about anything I really set my mind to do”) using a 4-point Likert scale (1 = *strongly agree* to 4 = *strongly disagree*). Higher scores indicated more feelings of control over life circumstances. Mastery was given in the self-report questionnaire prior to participants receiving instructions on completing the 30-day diary.

#### Daily diary: Negative and positive affect

Each day, participants completed the positive affect and negative affect scale, which totaled 32 items, PANAS [27]. The Negative Affect scale consisted of 16 items that assessed a general dimension of aversive affective states, such as feeling distressed, sluggish, hostile, and sad. The Positive Affect scale consisted of 16 items that assessed a general dimension of uplifting or positive affective states, such as feeling happy, relaxed, cheerful, and calm. Respondents indicated how often they had felt this way during the past 24 hours on a 5-point scale ranging from 1 (*very slightly/not at all*) to 5 (*extremely*). Consistent with the daily diary literature [16, 19], well-being is defined as levels of negative or positive affect on days when no negative or positive event was reported and emotional reactivity is defined as changes in negative or positive affect on days when a negative or positive event was reported.

#### Daily diary: Negative and positive daily events

During completion of the daily diary each night on the Tablet, participants answered questions pertaining to daily negative and positive events. The specific wording for daily negative events was, “*Think of the* *most stressful* *event that occurred today*, *even if it may not have been too stressful*. *Which category was this event in*?” The categories were spouse/partner, family, friends, work, finances, health, other, and no stressful event. For daily positive events, the specific wording was, “*Think of the* *most positive* *event that occurred today*, *even if it may not have been too positive*. *Which category was this event in*?” The categories were spouse/partner, family, friends, work, finances, health, other, and no positive event. From these items, we created two dichotomous variables, one for negative events and one for positive events, to indicate whether or not participants reported a negative or positive event during the course of the given day. If participants reported a negative or positive event occurring in the domains of spouse/partner, family, friends, work, finances, health, or other, then the negative or positive dichotomous variable was coded as a 1, with a 0 for days indicative of no negative or positive event.

Table 1 shows the breakdown of the frequency that each of the categories was reported during the course of the 30-day daily diary. We found that, on average, participants reported a negative event on 60% of the diary days, and, on average, participants reported a positive event on 79% of the diary days. The most frequent negative events reported were in the work, family, and other domains. The most frequent positive events reported were in the family, friend, and spouse/partner domains.

**Table 1:** Frequency of negative and positive daily events.

	Negative Events		Positive Events	
Event Domain	Observations	%	Observations	%
None	2,031	40	1,084	21
Spouse/partner	393	8	726	14
Family	541	11	1,138	22
Friend	198	4	806	16
Work	786	15	533	11
Finances	314	6	136	3
Health	374	7	200	4
Other	450	9	455	9

### Statistical Analysis

#### Multilevel logistic regression model

The first set of analyses focused on the extent to which childhood trauma was associated with differences in reports of a daily negative and positive event. Ultimately, our interest was in determining whether childhood trauma increased or decreased one’s likelihood of experiencing a negative or positive event over the course of the day. To do so, we used a multilevel logistic regression model, such that the log odds of the probability of reporting a negative or positive event was modeled as the outcome and childhood trauma was included as a person-level predictor. Models were estimated using SAS PROC GLIMMIX[59].

#### Multilevel linear regression model

The first set of analyses focused on the extent to which childhood trauma was associated with differences in reports of a daily negative and positive event. Ultimately, our interest was in determining whether childhood trauma increased or decreased one’s likelihood of experiencing a negative or positive event over the course of the day. To do so, we used a multilevel logistic regression model, such that the log odds of the probability of reporting a negative or positive event was modeled as the outcome and childhood trauma was included as a person-level predictor. Models were estimated using SAS PROC GLIMMIX [59].

#### Multilevel linear regression model

In a second set of analyses, we estimated a multilevel linear regression model [60] to examine whether childhood trauma moderated emotional reactivity to daily negative and positive events. Models were specified as

$${\text{WB}}_{ti}=\beta_{0i}\;+\beta_{1i}\;\left(negative\;event_{ti}\right)+\beta_{2i}\;\left(positive\;event_{ti}\right)+\;e{\;}_{ti}$$ (1)

where person *i*’s level of well-being (either negative affect or positive affect) at day *t*, WB_*ti*_, is a function of an individual-specific intercept parameter that represents levels of negative affect or positive affect on days when no negative or positive event was reported, β_0*i*_; an individual-specific emotional reactivity slope parameter, β_1*i*_, that captures rates of change in the outcome on days when a negative event was reported; an individual-specific emotional responsiveness slope parameter, β_2*i*_, that captures rates of change in the outcome on days when a positive event was reported and residual error, *e*_*ti*_.

Following standard multilevel modeling procedures [60], individual-specific intercepts and slopes (βs from the Level 1 model given in Eq 1) were modeled as the Level 2 model where between-person differences were estimated (i.e., variance parameters) and are assume to be normally distributed, correlated with each other, and uncorrelated with the residual errors, *e*_*ti*_. The expanded model that included childhood trauma, mastery, and socio-demographics took the form

$$\begin{array}{l} \begin{array}{l} \beta_{0i}=\gamma_{00}+\;\gamma_{01}({\text{childhood trauma}}_{i})+\;\gamma_{02}({\text{mastery}}_{i})\\ \;+\;\gamma_{03}({\text{childhood trauma}}_{i}\;{\text{x mastery}}_{i})\;+\;\gamma_{04}({\text{age}}_{i})+\;\gamma_{05}({\text{gender}}_{i})\\ \;+\;\gamma_{06}({\text{education}}_{i})+u_{0i} \end{array}\\ \begin{array}{l} \;\beta_{1i}=\gamma_{10}+\;\gamma_{11}({\text{childhood trauma}}_{i})+\;\gamma_{12}({\text{mastery}}_{i})\\ \;+\;\gamma_{13}({\text{childhood trauma}}_{i}\;{\text{x mastery}}_{i}) \end{array}\\ \begin{array}{l} \;\beta_{2i}=\gamma_{20}+\;\gamma_{21}({\text{childhood trauma}}_{i})+\;\gamma_{22}({\text{mastery}}_{i})\\ \;+\;\gamma_{23}({\text{childhood trauma}}_{i}\;{\text{x mastery}}_{i}) \end{array} \end{array}$$ (2)

All models were estimated using SAS PROC MIXED [61], with incomplete data accommodated under missing at random assumptions at the within- and between-person levels [62].

## Results

In a first step, we used the data from the 30-day daily diaries to create two aggregate measures of both negative affect and positive affect, namely a mean and standard deviation. The mean score represents one’s overall levels of negative affect and positive affect and the standard deviation score represents one’s fluctuations in negative affect and positive affect over the course of the 30-days. Table 2 shows the descriptive statistics for the variables included in the present study. The correlations from Table 2 suggest that reporting more childhood trauma was associated with overall higher levels of negative affect (*r* = .16, *p* <. 05) and lower levels of positive affect (*r* = −.21, *p* <. 05), in addition to greater variability in both negative affect (*r* = .21, *p* <. 05) and positive affect (*r* = .23, *p* <. 05). We also examined the association between childhood trauma and daily mean in negative and positive affect and variability in negative and positive affect using regression models. In the regression models, daily mean and variability in negative affect and positive affect were regressed on childhood trauma. We found substantively similar findings in that higher levels of childhood trauma were associated with higher negative affect, lower positive affect, and more variability in negative affect and positive affect. It is also worth noting the correlation between childhood trauma and mastery, *r* = −.24, *p* <. 05, suggesting that higher levels of childhood trauma is associated with reporting lower levels of mastery in midlife.

**Table 2:** Means, standard deviations, and intercorrelations among the constructs included in the study.

Construct	*M*	*SD*	1	2	3	4	5	6	7	8	9
1. Childhood trauma	1.68	0.86	–								
2. Mastery	3.17	0.65	–.24*	–							
3. Age	53.48	7.45	–.04	–.04	–						
4. Gender	0.54	0.50	.12	.004	–.07	–					
5. Education	0.75	0.43	–.03	.09	.19*	.05	–				
6. Mean negative affect	1.26	0.35	.16*	–.24*	–.02	–.11	.07	–			
7. Mean positive affect	3.13	0.80	–.21*	.23*	.21*	–.10	.05	–.21*	–		
8. SD negative affect	0.25	0.18	.21*	–.22*	–.09	.01	.08	.72*	–.19*	–	
9. SD positive affect	0.50	0.23	.23*	.05	–.08	.12	–.01	.10	–.20*	.35*	–

*N* = 191.

*p <.05.

To examine the data further, we created two groups, low and high levels of childhood trauma, based on a median split of the CTQ and examined whether there were significant differences in the study variables of interest. Table 3 shows that individuals who reported high levels of childhood trauma, on average, were more likely to report lower levels of mastery, more negative affect, less positive affect, and more variability in both negative and positive affect, but the two groups did not differ based on age, gender, and education. These preliminary results suggest that childhood trauma is associated reporting lower overall levels of and more fluctuations in well-being from day-to-day.

**Table 3:** Examining differences on key study variables based on low versus high levels of childhood trauma.

	Low Childhood Trauma (*N* = 124)		High Childhood Trauma (*N* = 67)	
	*M*	(*SD*)	*M*	(*SD*)
Measures				
Childhood Trauma	1.19	(0.20) _a_	2.57	(0.87) _b_
Mastery	3.29	(0.58) _a_	2.95	(0.71) _b_
Age	54.02	(7.54) _a_	52.48	(7.25) _a_
Gender	0.50	(0.50) _a_	0.62	(0.49) _a_
Education	0.76	(0.43) _a_	0.75	(0.44) _a_
Mean negative affect	1.22	(0.33) _a_	1.33	(0.38) _b_
Mean positive affect	3.25	(0.81) _a_	2.92	(0.75) _b_
SD negative affect	0.23	(0.18) _a_	0.29	(0.17) _b_
SD positive affect	0.46	(0.21) _a_	0.56	(0.24) _b_

*N* = 191. Median split was done on childhood trauma questionnaire. Subscripts that differ between columns are statistically significant at *p* <.05.

### Linking Childhood Trauma to Daily Negative and Positive Events

#### Reports of daily negative and positive events

Table 4 shows our results from a series of analyses examining whether childhood trauma was associated with the likelihood of experiencing negative and positive events each day. Focusing on daily negative events, we found that childhood trauma was associated with an increased likelihood of reporting a negative daily event (Odds Ratio = 1.43, 95% confidence interval: 1.06, 1.94). This suggests that each one unit increase in childhood trauma is associated with a 43% increased likelihood of reporting a daily negative event on a given day. To examine this finding further, we divided our sample into two groups, people with higher levels of childhood trauma (+1 *SD*; *n* = 30) versus lower levels of childhood trauma (–1 *SD*; *n* = 64) and examined whether they differed in the percent of days where a daily negative event was reported. In comparing the two groups, we found that the high childhood trauma group, on average, experienced daily negative events on 66% of the diary days, whereas the lower childhood trauma group, on average, experienced negative events on 50% of the diary days. The inclusion of mastery and socio-demographics rendered the effect of childhood trauma no longer significant. Predictors that were associated with an increased likelihood of a daily negative event were lower mastery and more years of education. Focusing on daily positive events, childhood trauma was not associated with engagement in daily positive events (Odds Ratio = 0.78, 95% confidence interval: 0.53, 1.18). Attaining more years of education was associated with increased likelihood of engaging in daily positive events.

**Table 4:** Examining whether childhood trauma is associated with reporting a daily negative and positive event.

	Negative Events						Positive Events			
	OR	[95% CI]	OR	[95% CI]			OR	[95% CI]	OR	[95% CI]
Fixed effects										
Childhood trauma	1.43*	[1.06, 1.94]	1.20		[0.89, 1.62]		0.78	[0.53, 1.18]	0.70	[0.46, 1.07]
Mastery			0.37*		[0.25, 0.55]				0.74	[0.41, 1.31]
Childhood trauma x mastery			1.13		[0.77, 1.66]				1.01	[0.59, 1.74]
Age			0.97		[0.94, 1.00]				1.02	[0.97, 1.07]
Gender			0.90		[0.56, 1.47]				1.35	[0.67, 2.75]
Education			2.59*		[1.45, 4.61]				3.23*	[1.41, 7.41]

*p <.05.

#### Emotional reactivity to daily negative and positive events

Table 5 shows results from our multilevel linear regression model that examined whether childhood trauma moderated emotional reactivity to daily negative and positive events. First, we found that within-person daily negative events resulted in increases in negative affect (γ_10_ = 0.17, *p* <. 05) and decreases in positive affect (γ_10_ = −0.15, *p* <. 05) and that presence of a positive event was associated with decreases in negative affect (γ_20_ = −0.07, *p* <. 05) and increases in positive affect (γ_20_ = 0.38, *p* <. 05). Childhood trauma was a significant predictor of the within-person daily negative event effect, such that reporting higher levels of childhood trauma was associated with stronger increases in negative affect (γ_11_ = 0.03, *p* <. 05) and stronger declines in positive affect (γ_11_ = −0.07, *p* <. 05) on negative event days. Similarly, childhood trauma was a significant predictor of the within-person daily positive event effect, such that reporting more childhood trauma was associated with stronger increases in positive affect (γ_21_ = 0.11, *p* <. 05).^2^ In follow-up analyses, we examined whether emotional reactivity to stressful events differed by the source of type of event (spouse/partner, family, friends, work, finances, health, and other). For daily negative events, more childhood trauma was associated with stronger declines in well-being for negative events centered on friends and health. For daily positive events, more childhood trauma was associated with stronger increases in well-being for positive events centered on spouse/partner, family, friends, work, and other.

**Table 5:** Examining emotional reactivity in negative affect and positive affect as a function of daily negative and positive events.

	Negative Affect		Positive Affect	
	Estimate	(*SE*)	Estimate	(*SE*)	
Fixed effects				
Intercept (no negative or positive event), γ_00_	1.27*	(0.03)	2.93*	(0.06)
Childhood Trauma, γ_01_	0.06	(0.03)	–0.22*	(0.07)
Negative Event, γ_10_	0.17*	(0.01)	–0.15*	(0.02)
Negative Event x Childhood Trauma, γ_11_	0.03*	(0.01)	–0.07*	(0.02)
Positive Event, γ_20_	–0.07*	(0.01)	0.38*	(0.02)
Positive Event x Childhood Trauma, γ_21_	0.001	(0.01)	0.11*	(0.02)
Random effects				
Intercept	0.11*	(0.01)	0.58*	(0.06)
Residual	0.09*	(0.002)	0.28*	(0.01)

Intraclass correlction (ICC): Positive affect = .672; Negative affect = .568.

*p <.05.

**Fig 1:**
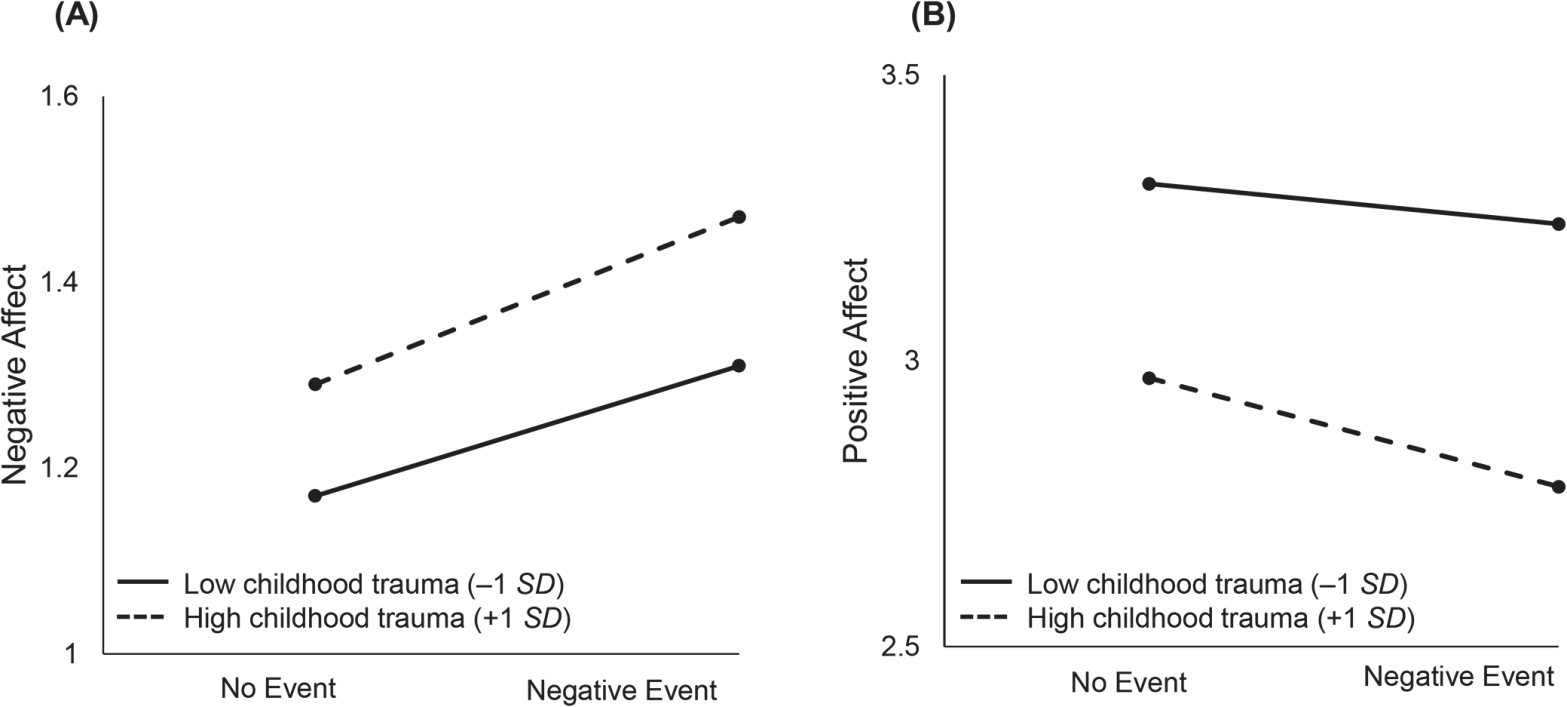
Illustrating the moderating effect of childhood trauma on within-person daily negative events. Compared to participants with lower levels of childhood trauma, participants with higher levels of childhood trauma were more likely to report more negative affect and less positive affect on non-event days (left part of A and B in Fig 1). Stronger changes or steeper slopes in negative and positive affect as a function of daily negative events is indicative of people with higher levels of childhood trauma being more emotionally reactive to daily negative events.

Fig 1 graphically illustrates the moderating effect of childhood trauma on within-person daily negative events. Compared to participants with lower levels of childhood trauma, participants who experienced more childhood trauma were more likely to report more negative affect and less positive affect on non-event days (left part of A and B in Fig 1). We see that stronger changes or steeper slopes in negative and positive affect as a function of daily negative events is indicative of people with childhood trauma being more emotionally reactive to daily negative events.

**Fig 2:**
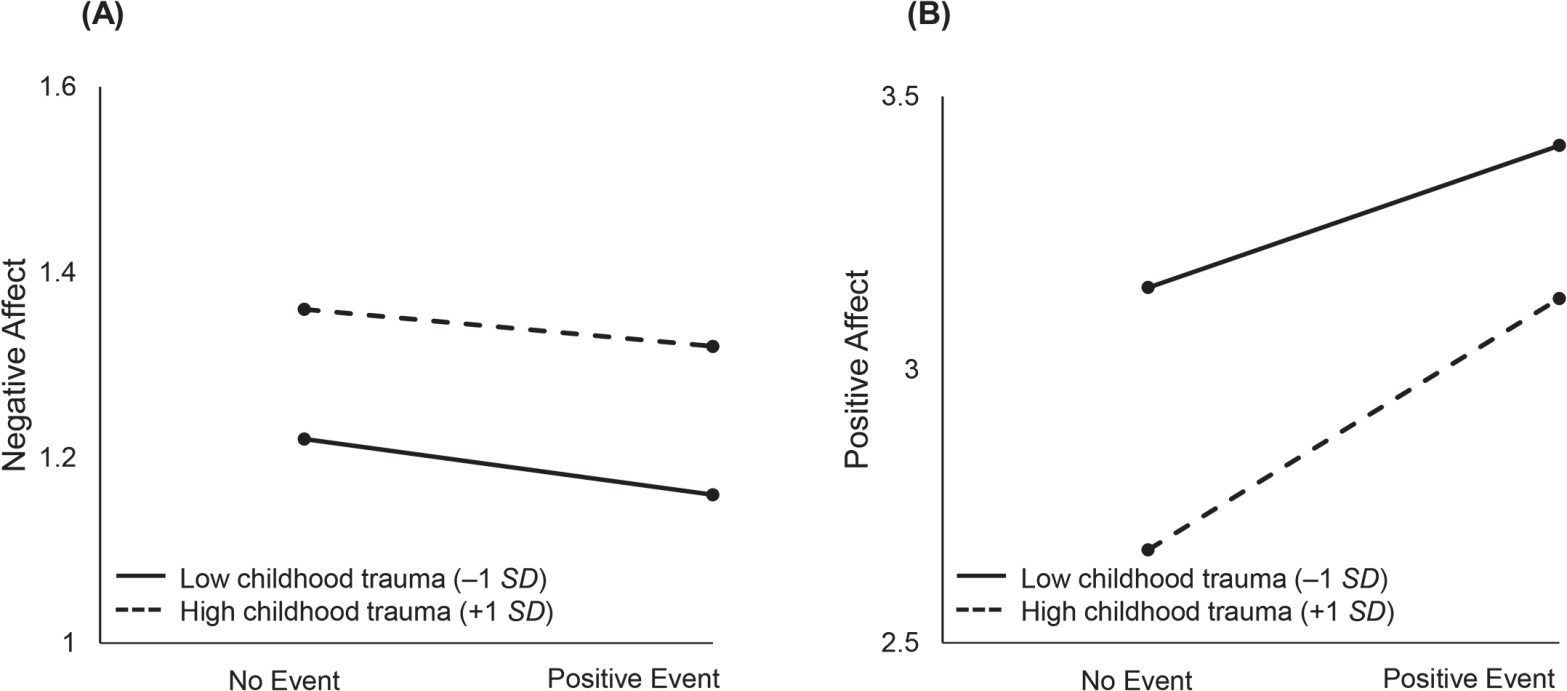
Illustrating the moderating effect of childhood trauma on within-person daily positive events. On days where a positive event was not reported, participants with higher levels of childhood trauma reported higher negative affect and lower positive affect (left part of A and B in Fig 2). People with higher levels of childhood trauma were more likely to show a stronger increase or boost in positive affect on days when a positive event was reported.

Fig 2 graphically illustrates the moderating effect of childhood trauma on within-person daily positive events. On days that a daily positive event was reported, people reporting higher levels of childhood trauma were more likely to show a stronger increase or boost in positive affect, suggesting that childhood trauma is associated with being more emotionally responsive to daily positive events.

**Fig 3:**
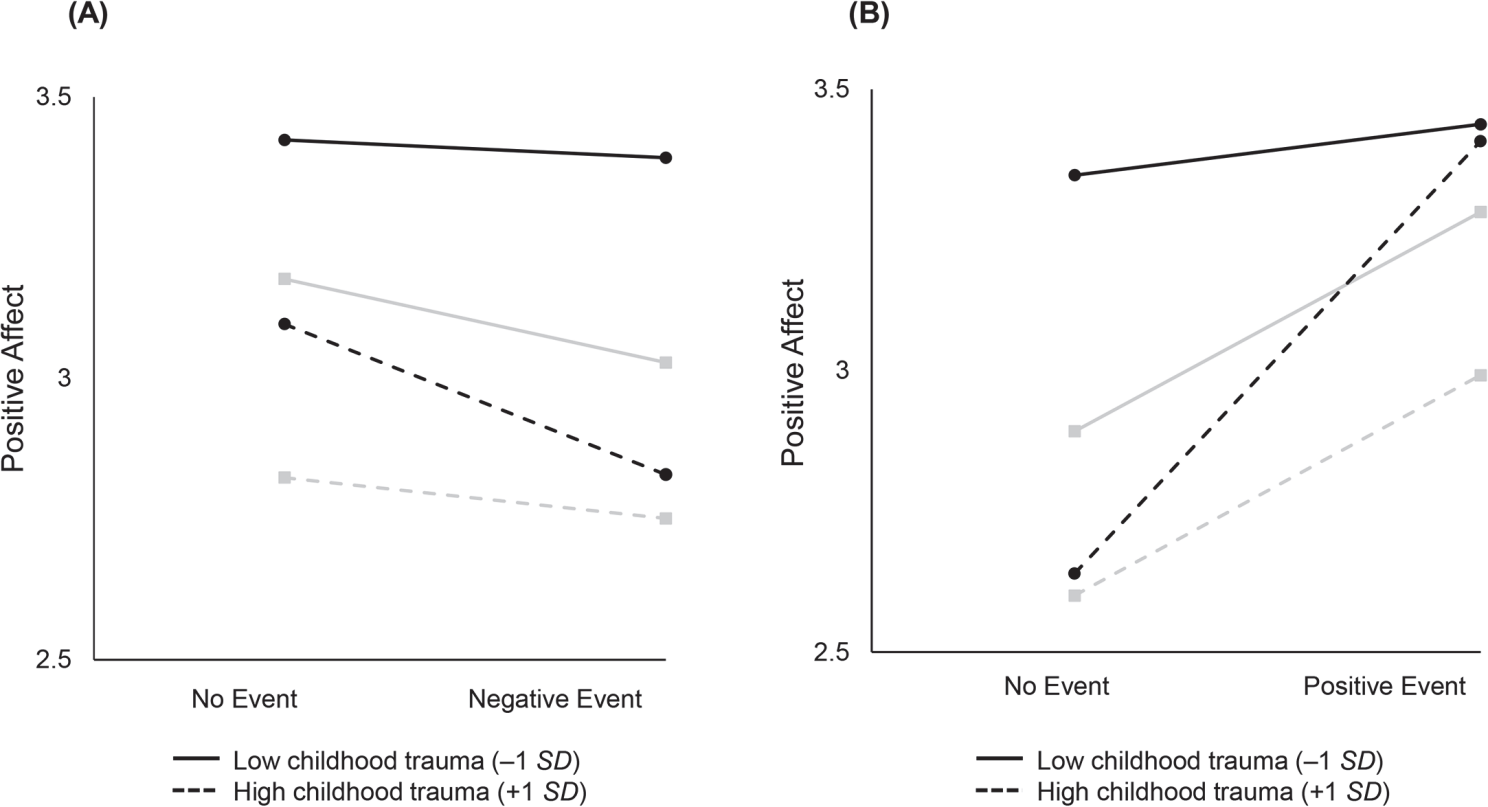
Illustrating the three-way interaction among within-person daily negative and positive events and between-person childhood trauma and mastery. Part A of Fig 3 shows that higher levels of childhood trauma and mastery is associated with stronger decreases in positive affect on days when confronted with a negative event (dotted line, circle end points). Part B of Fig 3 shows that higher levels of childhood trauma and mastery is associated with stronger increases in positive affect on days when confronted with a positive event (dotted line, circle end points). High mastery is represented by circle end points and black lines, whereas low mastery is represented by square end points and gray lines.

In a subsequent model, we included mastery and socio-demographics to examine whether mastery up- or down-regulated emotional reactivity to daily negative and positive events. Table 6 shows results from these analyses. We found that in the context of high childhood trauma, mastery increased one’s emotional reactivity to daily negative (negative affect: γ_13_ = 0.04, *p* <. 05; positive affect: γ_13_ = −0.003, *p* >. 05) and positive (negative affect: γ_23_ = −0.04, *p* <. 05; positive affect: γ_23_ = 0.12, *p* <. 05) events. Reporting more mastery in the context of high childhood trauma was associated with stronger increases in negative affect on days where a negative event was reported and stronger increases in positive affect on days when a positive event was reported. Fig 3 graphically illustrates the three-way interaction among within-person daily negative and positive events and between-person childhood trauma and mastery. Part A of Fig 3 shows that high childhood trauma and high mastery is associated with stronger decreases in positive affect on days when confronted with a negative event (dotted line, circle end points). Part B of Fig 3 shows that high childhood trauma and high mastery is associated with stronger increases in positive affect on days when confronted with a positive event (dotted line, circle end points). Our findings suggest that for people who experienced childhood trauma, mastery increases one’s sensitivity to their daily event context.

**Table 6:** Examining emotional reactivity in negative affect and positive affect as a function of daily negative and positive events: The role of mastery.

	Negative Affect		Positive Affect	
	Estimate	(*SE*)	Estimate	(*SE*)
Fixed effects				
Intercept (no negative or positive event), γ_00_	1.27*	(0.03)	2.92*	(0.06)
Childhood trauma, γ_01_	0.05	(0.03)	–0.20*	(0.07)
Age, γ_02_	–0.001	(0.003)	0.02*	(0.01)
Gender, γ_03_	–0.09	(0.05)	–0.11	(0.11)
Education, γ_04_	0.07	(0.06)	–0.06	(0.13)
Mastery, γ_05_	–0.15*	(0.04)	0.25*	(0.09)
Childhood trauma x mastery, γ_06_	0.01	(0.04)	–0.11	(0.09)
Negative event, γ_10_	0.18*	(0.01)	–0.16*	(0.02)
Negative event x childhood trauma, γ_11_	0.04*	(0.01)	–0.10*	(0.02)
Negative event x mastery, γ_12_	0.01	(0.02)	–0.03	(0.03)
Negative event x childhood trauma x mastery, γ_13_	0.04*	(0.02)	–0.003	(0.03)
Positive event, γ_20_	–0.08*	(0.01)	0.41*	(0.03)
Positive event x childhood trauma, γ_21_	–0.001	(0.01)	0.15*	(0.03)
Positive event x mastery, γ_22_	0.08*	(0.02)	0.01	(0.04)
Positive event x childhood trauma x mastery, γ_23_	–0.04*	(0.02)	0.12*	(0.03)
Random effects				
Intercept	0.11*	(0.01)	0.52*	(0.06)
Residual	0.09*	(0.002)	0.29*	(0.01)

Intraclass correlction (ICC): Positive affect = .672; Negative affect = .568.

*p <.05.

## Discussion

The objective of this study was to examine social-emotional regulation as one potential pathway linking childhood trauma to poorer health in midlife and beyond. We were able to test multiple components of social-emotional regulation using data from a 30-day daily diary of participants in midlife. We found that childhood trauma was associated with poorer overall levels of and greater fluctuations in daily well-being. Participants who reported higher levels of childhood trauma were more likely to also report stressors each day but their rate of reporting daily positive events did not differ. Furthermore, individuals with higher levels of childhood trauma were more emotionally reactive to daily negative and positive events, evidenced by stronger declines in well-being as a function of negative events and stronger increases in well-being with positive events (e.g., increased sensitivity to context, indicative of differential susceptibility). Lastly, mastery was not protective against decreases in well-being following negative events, but actually also increased one’s emotional reactivity to negative and positive events for people with childhood trauma. Our findings demonstrate how early life experiences still affect daily life in midlife and beyond. Furthermore, our results point to childhood trauma likely leading to poorer health in midlife through disturbances in the patterns of everyday life events and responses to those events.

### Pathways Linking Childhood Trauma to Health

Childhood trauma was associated with lower overall levels of and higher variability in daily well-being, which are both important constructs to take into consideration. How daily well-being is experienced likely operates through our daily behaviors and health practices that have subsequent effects on physiological processes that underlie the health effects of levels and variability in well-being [63]. For example, Ong and colleagues [64] found that sleep disturbance was associated with higher daily variability in positive affect in a mid-life sample, even though stable elevations in positive affect were associated with better sleep quality. Less consistency in daily well-being has also been tied to inflammatory markers associated with ill health [65].

A second component from the daily diaries ascertaining whether those reporting childhood trauma were also more likely to report daily negative and positive events; there was an association with stressful events but not positive experiences. The findings pertaining to average number of days reporting a daily stressor when contrasting high versus low levels of childhood trauma are startling. Our results indicate that over the course of 30 days, those reporting high levels of childhood trauma (+1 *SD*) averaged one or more negative events on 20 days compared with an average of 15 days for those reporting low levels of childhood trauma (–1 *SD*). In extrapolating these numbers to over the course of a year, this results in 60 more days, on average, with daily negative events than those with little or no abuse in their childhoods. Evidence from other studies suggests that these small stressors can have cumulative effects on health and mental health in midlife and beyond [16–17, 19].

#### Emotional reactivity to daily negative events

Our findings that childhood trauma was associated with greater emotional reactivity to daily negative events is consistent with previous research on adolescents [31–33]. These vulnerabilities to daily life stressors have been linked to long-term consequences in a number of recent studies [24, 64, 66, 67] and may be driven by underlying physiology. For example, several underlying physiological changes that occur as a result of reactivity to daily negative events include activation of the sympathetic and parasympathetic nervous system [68]. These physiological pathways were not testable in our dataset, but should be explored in future research [69]. In sum, childhood trauma may lead to daily life being, on average, more *stressful* and *unpleasant* and over years and decades, this likely accumulates to shape the course of health in midlife and beyond.

#### Emotional reactivity to daily positive events

Childhood trauma was associated with greater increases in well-being on days that notable positive events occurred. This finding, coupled with showing that childhood trauma was associated with emotional reactivity to daily negative events, points to the possibility that childhood trauma makes individuals more sensitive to their daily context, otherwise known as differential susceptibility. Broadly speaking, differential susceptibility is defined as contexts through which individuals suffer with negative events and flourish with positive events [35, 70–71]. Childhood adversity may differentially boost well-being through greater responsiveness to lasting changes that have a positive valence. Early life adversity can lead to higher levels of inflammation (13), but positive emotions can offset increases in inflammation and improve immune functioning [72]. It is important to note that greater responsivity to positive events does not mean that those with greater childhood adversity had occasions when they were happier than those without those past experiences. As Fig 2 shows they approach but do not surpass levels of well-being of those without childhood trauma. Greater sensitivity, though, does suggest that this population would respond to interventions designed to help them attain more positive social experiences.

### Mastery as a Key Resilience Factor for Confronting Childhood Trauma

Before discussing the moderating role of mastery in the context of emotional reactivity to daily negative and positive events for those with childhood trauma, we note that childhood trauma and mastery were moderately correlated, *r* = −.24, suggesting that childhood trauma is associated with reporting lower levels of mastery. There is limited research in the childhood antecedents of mastery and this finding suggests that childhood trauma sets individuals on a developmental course of lower levels of mastery.

Our findings on the effects of mastery did not fully match our hypotheses. Mastery was associated with a stronger increase in well-being on days when a positive event was reported. However, mastery was not always protective against declines in well-being, and even in fact exacerbated emotional reactivity to negative events in some cases. For people with high levels of childhood trauma, reporting more mastery beliefs were associated with greater decreases in well-being with daily negative events. Our findings suggest that mastery, rather being a positive personality feature across the board, increases one’s sensitivity to both good and bad events in everyday life.

There are several plausible reasons for this finding. First, mastery beliefs likely constitute something different for people with high levels of childhood trauma. Individuals who experienced childhood trauma and report more mastery may have John Henryism, a term coined by James and colleagues as signifying someone who believes they can do all and need no help, when in fact with difficult stressors this is less adaptive [73]. Similarly, those reporting high levels of mastery may be low on other important resources for managing stressors such as perceived social support. Therefore, these individuals may be too self-sufficient and fearful of relying on others. Second, daily stressors are typically events, experiences, and interactions that are largely uncontrollable [74] and this could be compounded for people with childhood trauma and high in mastery. Being high on mastery may result in viewing the world in general as having control over life circumstances, but when encountered with uncontrollable daily events, this clashes with one’s expectations, resulting in stronger declines in well-being as a function of daily stressors.

### Resilience-Promoting Interventions

Our findings that higher levels of childhood trauma are associated with increased sensitivity to daily context (i.e., differential susceptibility) may result in people being more *sensitive* or *responsive* to interventions. There are various routes researchers can take to intervene for better health in midlife and beyond for people with high levels of childhood trauma. First, researchers can focus on increasing levels of mastery. The focus of interventions targeting mastery could be controllability and uncontrollability of daily life events, particularly, negative events. People need to acknowledge that certain daily events, whether they be negative or positive, may not be controllable. For example, Zautra and colleagues in a 25-day daily intervention focused on increasing mastery beliefs through daily phone calls, with messages that focused on identifying positive and negative experiences that were under their control, and encouraging participants to take action to improve their daily lives [75]. They found that, although mastery beliefs remained relatively stable over the course of the intervention, the intervention resulted in significant improvements in mental health [75].

Second, social relationship interventions have broad-based applicability and could help people with childhood trauma reduce the toxic effects of stress on health and overall functioning [76–77]. Social Intelligence concepts have been embedded within the social and behavioral sciences literature for some time [78], and in current writings, these concepts refers to a keen awareness of the value of sustainable social connections, the ability to take another’s perspective, and the capacity to engage fully in satisfying relationships [79–80]. Focusing on the plasticity of social relationships through social intelligence training has the potential to benefit people with high levels of childhood trauma through modifying key social cognitions regarding social engagement, and enhance efficacy expectations regarding performance in social situations [81]. The approach extends beyond cognitive models and behavioral principals to include attention to evidence of barriers to social-emotional development from adverse experiences in childhood and adult life [76].

### Limitations and Conclusion

We note several limitations. First, our measure of childhood trauma was a retrospective self-report and people do not always report their childhood experiences, reliably. Prior studies have used the CTQ in clinical populations to obtain reliable accounts of emotional, sexual, or physical abuse in childhood [58]. The CTQ measure complements research from previous studies that have focused on childhood misfortune [3]. Second, our sample was drawn from the Phoenix metropolitan area, and though it may be fairly representative of middle-aged residents in the southwestern United States, its representativeness of midlife in other regions is unknown. Future research is needed on examine the extent to which similar associations are found in other samples of people in midlife, as well as those in young adulthood and old age. It may be that emotional reactivity to stressors is strongest for those individuals who are in young adulthood and experienced childhood trauma, as compared to people in older ages. Third, it is likely that daily well-being, and reports of and reactivity to daily negative and positive events have physiological consequences for subsequent effects on health. This is in line with research showing that stressors are associated physiological effects [68, 82–83] and future research is warranted that focuses on sympathetic and parasympathetic systems to determine how the physiological changes mediate the effects of stress to eventual health declines.

Early life adversity in the form of childhood trauma has the potential to shape the course of development in midlife and beyond. We explored the extent to which daily social-emotional regulation links childhood trauma to premature declines in health, with a 30-day daily diary that allowed for probing multiple components of social-emotional regulation, such as daily level and variability in well-being, as well as experience of and reactivity to daily negative and positive events. Our findings suggest that one potential pathway linking childhood trauma to poorer overall health in midlife and beyond is through disturbances in the patterns of everyday events and responses to those events. We urge future inquiry examining specific mechanisms underlying these relationships.

## Supporting Information

S1 Dataset Dataset for analyses reported in this manuscript. (ZIP)
